# Conditions Under Which Glutathione Disrupts the Biofilms and Improves Antibiotic Efficacy of Both ESKAPE and Non-ESKAPE Species

**DOI:** 10.3389/fmicb.2019.02000

**Published:** 2019-08-30

**Authors:** Theerthankar Das, Denis Paino, Arthika Manoharan, Jessica Farrell, Greg Whiteley, Frederik H. Kriel, Trevor Glasbey, Jim Manos

**Affiliations:** ^1^Department of Infectious Diseases and Immunology, Central Clinical School, Faculty of Medicine and Health, The University of Sydney, Sydney, NSW, Australia; ^2^Whiteley Corporation, North Sydney, NSW, Australia; ^3^School of Medicine, Western Sydney University, Campbelltown, NSW, Australia; ^4^Whiteley Corporation, Tomago, NSW, Australia

**Keywords:** glutathione, biofilm, *Acinetobacter baumannii*, antibiotic resistance, thiol antioxidants

## Abstract

Bacterial antibiotic resistance has increased in recent decades, raising concerns in hospital and community settings. Novel, innovative strategies are needed to eradicate bacteria, particularly within biofilms, and diminish the likelihood of recurrence. In this study, we investigated whether glutathione (GSH) can act as a biofilm disruptor, and enhance antibiotic effectiveness against various bacterial pathogens. Biological levels (10 mM) of GSH did not have a significant effect in inhibiting growth or disrupting the biofilm in four out of six species tested. However, exposure to 30 mM GSH showed >50% decrease in growth for all bacterial species, with almost 100% inhibition of *Streptococcus pyogenes* and an average of 94–52% inhibition for *Escherichia coli*, Methicillin-resistant *Staphylococcus aureus* (MRSA) and Methicillin-sensitive *S. aureus* (MSSA) and multi-drug resistant *Acinetobacter baumannii* (MRAB) isolates, respectively. *Klebsiella pneumoniae* and *Enterobacter* sp. isolates were however, highly resistant to 30 mM GSH. With respect to biofilm viability, all species exhibited a >50% decrease in viability with 30 mM GSH, with confocal imaging showing considerable change in the biofilm architecture of MRAB isolates. The mechanism of GSH-mediated biofilm disruption is possibly due to a concentration-dependent increase in GSH acidity that triggers cleaving of the matrix components. Enzymatic treatment of MRAB revealed that eDNA and polysaccharides are essential for biofilm stability and eDNA removal enhanced amikacin efficiency. Combination of GSH, amikacin and DNase-I showed the greatest reduction in MRAB biofilm viability. Additionally, GSH alone and in combination with amikacin fostered human fibroblast cell (HFF-1) growth and confluence while inhibiting MRAB adhesion and colonization.

## Introduction

The spread of antibiotic resistance in the hospital environment and in community settings particularly where biofilms are involved, has spurred development of new strategies to design innovative therapeutics to curtail the spread of resistance. In 2016, a review commissioned by the UK government concluded that approximately 700,000 people die each year around the globe from antibiotic-resistant infections (O’Neill, 2016). In Australia, the burden of Healthcare-associated infections are estimated around 165,000 annually comprising bacterial associated urinary tract infections, blood stream infection, pneumonia, surgical site infections, respiratory Infections etc. (Mitchell et al., 2017). In addition, antibiotic resistance has a serious global economic impact. It has been projected that antibiotic resistance will cost US$100 trillion worldwide by 2050, with an estimated yearly death toll reaching 10 million during that period (O’Neill, 2015). Bacterial resistance is critically exacerbated through the extensive and unwarranted use of antibiotics in sectors including aged care, human and hospital usage, agriculture and food animal production. Current statistics suggest that India, China, United States, Russia, Brazil, and South Africa are the world leaders in per capita consumption of antibiotics (Van Boeckel et al., 2014). A report by Antimicrobial Use and Resistance in Australia (AURA) 2017 suggests extensive misuse of antibiotics is occurring in Australian hospitals e.g., in 2015, 40.5% of hospital in-patients were being prescribed an antimicrobial, with 21.9% of cases considered inappropriate and 23.3% of antimicrobial prescriptions were non-compliant with guidelines (AURA, 2017).

Bacteria in biofilm growth phase are principally responsible for an array of infections within hospital, general health care and community settings (Jamal et al., 2018). Bacterial self-produced extracellular molecules such as extracellular DNA (eDNA), polysaccharides, proteins and metabolites aids them to adhere and colonize on surfaces and forms structurally stable biofilm matrix (Flemming and Wingender, 2010; Das et al., 2011, 2013). Bacteria embedded in its matrix endure substantial physical stress (shear) and significantly, higher chemical stress than planktonic bacteria, including stress from antibiotics, antiseptics and detergents (Lewis, 2001; Mahzounieh et al., 2014; Karimi et al., 2015; Peterson et al., 2015; Wilton et al., 2016; Clayton and Thien-Fah, 2017). Bacteria in its biofilm state also resist host immune response and triggers infection (Domenech et al., 2013; Watters et al., 2016). Due to the slow diffusion of the antibiotics through biofilm matrix, the presence of biofilms represents a big eradication challenge compared to planktonic bacteria (Lewis, 2001; Wilton et al., 2016; Clayton and Thien-Fah, 2017). Biofilm-associated infections in a host lead to changes in various vital biological systems such the host immune response. They also lead to depletion of antioxidants such as glutathione (GSH), the master antioxidant in all mammalian cells. The depletion of GSH has been directly linked to an increase in pathogenicity of infection (Ristoff et al., 2001; Ghezzi, 2011). GSH depletion during bacterial infection induces oxidative stress in host cells and consequently inhibits cell growth, thus triggering cell death (Ristoff et al., 2001; Ghezzi, 2011). GSH is a thiol (-SH) based tripeptide antioxidant, and in protecting mammalian cells against oxidative stress, it aids in cell cycle regulation and growth as well as maintenance of redox homeostasis (Forman et al., 2009). In our previous study, we elucidated GSH’s significant role in neutralizing the cytotoxicity of the *Pseudomonas aeruginosa* virulence factor pyocyanin, and facilitating the growth of human lung epithelial cells (Das et al., 2017). In *P. aeruginosa* biofilms, GSH directly interacts with pyocyanin and modulates its structure to inhibit pyocyanin intercalation with DNA and biofilm integration and in this way disrupts the biofilm matrix and enhances antibiotic efficiency (Das et al., 2015; Klare et al., 2016).

Amongst the ESKAPE (*Enterococcus faecium*, *Staphylococcus aureus, Klebsiella pneumoniae, Acinetobacter baumannii, Pseudomonas aeruginosa*, and *Enterobacter* species) pathogens, prominent biofilm forming bacteria include *S. aureus* and *A. baumannii. A. baumannii*, a Gram-negative emerging pathogen, has garnered attention in recent years due to its inherent multidrug resistant (MDR) profile and pathogenicity, responsible for causing nosocomial infections in hospitalized patients, skin and soft tissue infections especially predominate in combat-associated wounds, morbidity, and mortality in weakened and critically ill patients (Joly-Guillou, 2005; Perez et al., 2007; Fishbain and Peleg, 2010). The World Health Organization (WHO) has classified *A. baumannii* as of critical importance alongside *P. aeruginosa* and the *Enterobacter* sp. whereas; *S. aureus* are classified as of high importance^1^.

In this study, we aimed to identify whether GSH plays a role in biofilm disruption in non-pyocyanin-expressing pathogenic species. These experiments included testing the effect of a combination treatment (CT) comprising GSH and an antibiotic of choice, on clinical strains. Strains included four bacterial species of the ESKAPE group of pathogens: *A. baumannii*, methicillin resistant *S. aureus* (MRSA) and methicillin sensitive *S. aureus* (MSSA), Enterobacter sp. (*E. cloacae* and *E. aerogenes*) and *K. pneumoniae* as well as the non-ESKAPE pathogens *Streptococcus pyogenes* and *Escherichia coli*, all clinical isolates sourced from Sydney hospital culture collections. Most of the bacterial isolates used in this study were strongly susceptible to ciprofloxacin, whereas the multi drug resistant *A. baumannii* (MRAB) isolates were sensitive only to amikacin. The study was also expanded in the case of *A. baumannii* to investigate the effect of enzymes on the efficiency of GSH plus amikacin (a triple combination therapy), in disrupting MRAB biofilms.

## Materials and Methods

### Bacterial Strains

Species used in this study were *S. aureus* (MRSA and MSSA), *S. pyogenes*, *A. baumannii* (MRAB), Enterobacter sp., *K. pneumoniae* and *E. coli*. Strains were obtained from Royal Prince Alfred Hospital, Concord Hospital and the High Risk Foot Service clinic at Liverpool Hospital, all located in Sydney, Australia. The Tissue Act (NSW, 1983)^2^ did not require the study to be reviewed or approved because: all species/strains were de-identified by the hospitals concerned prior to being gifted to us, all species/strains were from their historical culture collections, and they were not collected from patients as part of this study. Table 1 lists the species/strains, their source (hospital details) and antibiotic MIC’s. The ciprofloxacin MIC for as measured for each species was: [*S. aureus* MRSA and MSSA (=10 μg/ml); *S. pyogenes* (4 μg/ml); *Enterobacter* sp. (=0.5 μg/ml); *E. coli* (=0.5 μg/ml); *K. pneumoniae* (=10 μg/ml)], and the amikacin MIC was (4 μg/ml) for MRAB.

**TABLE 1:** Source and antibiotic profile of bacterial species/strains obtained from hospital culture collections.

	**Source**	**Antibiotic profile^∗^**					
**Bacterial species**		**Amk**	**Aug**	**Amx**	**Cip**	**Gen**	**Tob**
***Staphylococcus aureus* (MRSA)**							
MRSA-1 (Left leg wound)	Microbiology Department, RPAH, Sydney, NSW, Australia	NT	R	R	S	S	NT
MRSA-2 (Chin vesicle)	Microbiology Department, RPAH, Sydney, NSW, Australia	NT	R	R	S	S	NT
MRSA-3 (DFU)	The High-Risk Foot Service clinic, Liverpool hospital, Sydney, NSW, Australia	R	R	R	R	R	R
***Staphylococcus aureus* (MSSA)**							
MSSA-1 (Left elbow)	Microbiology Department, RPAH, Sydney, NSW, Australia	NT	S	R	S	S	NT
MSSA-2 (Right toe)	Microbiology Department, RPAH, Sydney, NSW, Australia	NT	S	R	S	S	NT
MSSA-3 (DFU)	The High-Risk Foot Service clinic, Liverpool hospital, Sydney, NSW, Australia	R	S	S	R	R	R
***Streptococcus pyogenes***							
SP-1 (left leg boil)	Microbiology Department, RPAH, Sydney, NSW, Australia	NT	S	S	S	NT	NT
SP-2 (head wound)	Microbiology Department, RPAH, Sydney, NSW, Australia	NT	S	S	S	NT	NT
SP-3 (skin wound)	Microbiology Department, RPAH, Sydney, NSW, Australia	NT	S	S	S	NT	NT
***Acinetobacter baumannii***							
MRAB-1 (Urine)	Microbiology Department, Concord Hospital, NSW, Australia	S	R	R	R	R	R
MRAB-2 (Catheter)	Microbiology Department, Concord Hospital, NSW, Australia	S	R	R	R	R	R
MRAB-3 (Skin)	Microbiology Department, Concord Hospital, NSW, Australia	S	R	R	R	R	R
MRAB-4 (Catheter)	Microbiology Department, Concord Hospital, NSW, Australia	S	R	R	R	R	R
***Enterobacter Species***							
ENTC-1 (Ear)	Microbiology Department, RPAH, Sydney, NSW, Australia	S	R	R	S	S	S
ENTC-2 (Wound)	Microbiology Department, RPAH, Sydney, NSW, Australia	S	R	R	S	R	R
ENTA-1 (Sternum)	Microbiology Department, RPAH, Sydney, NSW, Australia	S	R	R	S	S	S
***Escherichia coli***							
EC-1 (Drain fluid)	Microbiology Department, RPAH, Sydney, NSW, Australia	S	S	R	S	S	S
EC-2 (Wound)	Microbiology Department, RPAH, Sydney, NSW, Australia	S	R	R	S	S	S
EC-3 (Exit site)	Microbiology Department, RPAH, Sydney, NSW, Australia	S	S	S	S	S	S
***Klebsiella pneumoniae***							
KP-1 (Catheter urine)	Microbiology Department, RPAH, Sydney, NSW, Australia	S	R	R	S	S	S
KP-2 (Left hip wound)	Microbiology Department, RPAH, Sydney, NSW, Australia	S	I	R	I	R	S
KP-3 (Neck pus)	Microbiology Department, RPAH, Sydney, NSW, Australia	S	S	R	S	S	S

*RPAH, Royal Prince Alfred Hospital; DFU, diabetic foot ulcer; Antibiotics: Amk, amikacin; Aug, augmentin; Amx, amoxicillin; Cip, ciprofloxacin; Gen, gentamicin; Tob, tobramycin; NT, not tested; R, resistance; S, sensitive, and I, intermediate. Ciprofloxacin MIC: *S. aureus* MRSA and MSSA (=10 μg/ml); *S. pyogenes* (4 μg/ml); *Enterobacter* sp. (=0.5 μg/ml); *E. coli* (=0.5 μg/ml); *K. pneumoniae* (=10 μg/ml). Amikacin MIC for *A. baumannii* (4 μg/ml).*

### General Chemicals Used for This Study

Glutathione, Glutathione disulfide (GSSG), antibiotics (ciprofloxacin, amikacin, Augmentin, gentamicin and tobramycin), resazurin dye, crystal violet solution, Phosphate buffered saline (1 × PBS) and Hydrogen peroxide (H_2_O_2_) were all obtained from Sigma-Aldrich (Sydney, Australia). Tryptone Soy Broth (TSB) was obtained from Oxoid (Thermo Fisher Scientific, Australia), Enzymes: DNase-I was obtained from Invitrogen (Melbourne, Australia), α-Amylase from MP Biomedicals (NSW, Australia) and Proteinase K from Sigma-Aldrich (Sydney, Australia). Dulbecco’s Modified Eagle Medium (DMEM) and Fetal bovine serum (FBS) from Sigma-Aldrich (Sydney, Australia).

### Determining the Change in pH of TSB and 1 × PBS as a Function of GSH Concentration

Glutathione powder was weighed and directly dissolved into TSB (pH 7.18) and 1 × PBS (137 mM NaCl, 2.7 mM KCl and 10 mM phosphate, pH 7.41) solution at room temperature under sterile conditions (bio-safety cabinet) to give 10, 20, and 30 mM of GSH. The change in pH of TSB and 1 × PBS at different GSH concentrations was determined using a pH meter (Mettler-Toledo GmbH, Greifensee, Switzerland) calibrated with pH standards of 4.0 and 7.0 and the pH values of TSB and 1 × PBS as a function of GSH concentration reported in Table 2.

**TABLE 2:** Change in pH of TSB and PBS in presence of glutathione (GSH).

	**GSH concentration (mM)**	**pH**
Tryptone soy broth (TSB)	0	7.18 ± 0.08
	10	6.45 ± 0.1
	20	5.59 ± 0.11
	30	4.77 ± 0.05
Phosphate buffer saline (PBS)	0	7.41 ± 0.03
	10	5.51 ± 0.09
	20	3.89 ± 0.14
	30	3.21 ± 0.16

### Determining the Effect of GSH and Antibiotics on Planktonic Growth

All clinical isolates listed in Table 1 were grown in TSB medium for 24 h, at 37°C and 150 rpm. After growth, the planktonic cultures were harvested by centrifugation at 5000×*g* for 5 min at 10°C, followed by removal of the supernatant and resuspension of the bacterial pellet in TSB. The effect of GSH plus antibiotic on planktonically-grown isolates was determined by taking 250 μL (OD_600_ = 0.1 ± 0.02) of bacterial cell suspension into the wells of 96-well plates (Corning Corp. United States) and incubating for up to 48 h, at 37°C and 150 rpm. The bacterial growth media contained one of: 0, 10, 20, and 30 mM GSH, 0.25–10 μg/ml ciprofloxacin or 4–16 μg/ml amikacin (only for *A. baumannii*). Bacterial growth was measured at 48 h post-treatment by recording absorbance at OD_600__nm_ using a plate reader (Tecan infinite M1000 pro). The final increase in bacterial growth at 48 h was measured by subtracting the absorbance at 48 h from the absorbance at 0 h. Controls were untreated, and showing 100% growth, while tests measured percentage decrease in bacterial growth with respect to this control.

### Determining the Minimum Biofilm Inhibitory Concentration (MBC) of Amikacin on *A. baumannii*

All *A. baumannii* (MRAB isolates listed on Table 1) were grown in TSB medium for 24 h, at 37°C and 150 rpm. After growth, the planktonic cultures were adjusted to OD_600_ = 0.5 ± 0.05 in TSB. 200 μl of bacterial culture was then added to the wells of 96-well plates (Corning Corp. United States) and incubated at 37°C for 60 min at 150 rpm. After 60 min, the wells were gently washed once with 1 × PBS to remove any loosely adhered bacteria. 200 μL of TSB was then added, followed by further incubation at 37°C for 48 h and 150 rpm to initiate biofilm growth. In amikacin-treated groups, biofilms were grown in the presence of 4–18 μg/ml amikacin dissolved in TSB. After 48 h of incubation, the biofilms were washed once with 1 × PBS, followed by addition of 200 μL of 1 × PBS and 15 μL of a 0.05% w/v resazurin solution. Plates were then incubated for a further 24 h at 37°C and 150 rpm. Biofilm fluorescence intensity was then determined at Ex_544__nm_ and Em_590__nm_ (Tecan infinite M1000 pro, Australia). Amikacin-treated groups were compared for percentage decrease in biofilm viability to 1 × PBS-treated control wells showing 100% bacterial cell viability. The percentage increase in bacterial growth at 48 h was determined as mentioned above.

### Effect of GSH and Antibiotics on Biofilm Viability

Bacterial isolates were grown as described above. To initiate biofilm growth, planktonic bacteria were re-suspended in TSB and 250 μL of bacterial cell suspension (OD_600_ = 0.5 ± 0.05) was added into the wells of 96-well plates (Corning Corp. United States). Plates were incubated for 48 h, at 37°C and 150 rpm. After 48 h, the biofilm was washed once with 1 × PBS followed by treatment (24 h, 37°C, 150 rpm) as follows: Control (1 × PBS), 0.5–30 μg/mL ciprofloxacin – depending on MIC of bacterial strain, or 10 and 30 mM GSH; or a combination of ciprofloxacin + GSH. MRAB biofilms were treated with 4 μg/mL, 12 μg/mL, MBC (15–16 μg/mL) and 20 μg/mL amikacin, 10 and 20 μg/mL ciprofloxacin, 10 and 20 μg/mL Augmentin and 10 and 20 μg/mL tobramycin individually, or in combination with GSH. All antibiotic and GSH treatment solutions were prepared in 1 × PBS (pH 7.4).

After 24 h, treated biofilms were washed once with 1 × PBS followed by addition of 200 μL of 1 × PBS and 15 μL of a 0.05% w/v resazurin solution. Plates were then incubated for a further 24 h at 37°C and 150 rpm. After incubation, the fluorescence intensity of the biofilm was determined at Ex_544__nm_ and Em_590__nm_ (Tecan infinite M1000 pro, Sydney Australia). Test biofilms were compared for percentage decrease in biofilm viability to 1 × PBS-treated control wells showing 100% bacterial cell viability.

### MRAB Biofilm Biomass Quantification by Crystal Violet (CV) Staining

Multi-drug resistant *A. baumannii* biofilms were grown for 48 h in 96-well plates as described above. After 48 h, biofilms were washed once with 1 × PBS followed by incubation for 24 h, at 37°C, 150 rpm with: 4, 12, and 20 μg/mL amikacin or 10, 15, and 30 mM GSH or a combination of 30 mM GSH + 4 μg/mL amikacin whereas, controls were treated with 1 × PBS. All test solutions were prepared in 1 × PBS (pH 7.4). After 24 h, treated biofilms were washed once with 1 × PBS. The biofilm biomass attached to the wells was then stained with 200 μL 0.05% (w/v) CV and incubated a further 1 h at 37°C, 150 rpm. After incubation, cells were washed three times with 1 × PBS to remove excess CV stain. The pre-stained biofilm was then allowed to dry for 15 min at 37°C, after which it was dissolved using 80% v/v ethanol and transferred into new 96-well plate for biomass quantification at OD_550__nm_ using a Tecan plate reader (Infinite M1000 pro). Controls were treated with 1 × PBS and showed 100% growth, while tests measured the percentage decrease in biofilm biomass with respect to this control.

### Effect of Enzymatic Treatment on MRAB Biofilm Biomass

Multi-drug resistant *A. baumannii* biofilms were grown for 48 h in 96-well plates as described above. After 48 h, biofilms were washed once with 1 × PBS followed by treatment with different enzyme solutions prepared in 1 × PBS, pH 7.4, including: 5, 10, 20, and 40U DNase-I, 100, 200, 500 and 1000 μg/ml amylase and 50, 100, 200 and 500 μg/ml Proteinase K, for 24 h, at 37°C and 150 rpm, the control was treated with 1 × PBS alone. After 24 h, treated biofilms supernatant washed once with 1 × PBS. The biofilm biomass attached to the wells was then stained with 0.05% (w/v) CV and biofilm biomass quantified using the above protocol. Control group normalized as 100% biofilm biomass and enzyme-treated groups were compared with the control to measure percentage decrease in biomass.

### Analysis of MRAB Biofilm Architecture Using Confocal Laser Scanning Microscopy (CLSM)

To initiate biofilm growth, 500 μL MRAB-3 (OD_600_ = 0.5 ± 0.05) in TSB was added to microscope glass slides and incubated at 37°C in a static incubator for 48 h. After 48 h, biofilms were washed once with 1 × PBS and treated using one of the following: 30 mM GSH or 4 μg/mL amikacin individually or a combination of 30 mM GSH + 4 μg/mL amikacin. Enzymatic treatment included 40U DNase-I, 1000 μg/ml Amylase, 500 μg/ml Proteinase K and a combination of 40U DNase-I + 4 μg/mL amikacin or 40U DNase-I + 30 mM GSH. Finally, MRAB-3 biofilms were also subjected to a three-part combination treatment: 30 mM GSH + 40U DNase-I + 4 μg/mL amikacin. For control, MRAB-3 biofilms were treated with 1 × PBS. All treated biofilms were then incubated for 24 h at 37°C in a static incubator. After 24 h incubation, control and all treated biofilms were washed three times with 1 × PBS to remove any planktonic/loosely bound bacterial cells. Biofilms were then stained with a live/dead stain (Bacterial viability kit, Life Technologies Inc., United States) for 30 min in the dark and cells were then visualized by CLSM (Olympus FV1200, Australia) with Ex_473_ and _559__nm_ and Em_500_ and _637__nm_, for Syto-9 (green-live) and propidium iodide (red-dead) staining, respectively. ImageJ software was used to generate images and quantify percentage of live and dead biofilm cells for biovolume.

### Analysis of the DNA–GSH Interaction by Circular Dichroism

A Circular Dichroism (CD) Spectropolarimeter (Jasco 815, Easton, MD, United States) was used to investigate DNA-GSH reactions in a 1-mm path length quartz cuvette. dsDNA (calf thymus DNA-sodium salt Type 1 fibers, Sigma-Aldrich, Australia) and GSH stock solutions were prepared in sterile MilliQ water. To study the interaction, 200 ng/μl dsDNA incubated for 24 h at 37°C, 100 rpm, in either absence or presence of 1 mM GSH at intrinsic pH or 1 mM GSH at neutral pH (7.2). 300 μl aliquots pipetted into cuvettes and scanned by CD at 200–320 nm wavelength in a static condition at 25°C.

### Investigating Effect of GSH on dsDNA by Fluorometry

A total of 200 ng/ μl dsDNA was incubated for 24 h at 37°C, 100 rpm, in either presence or absence of 1 mM GSH at intrinsic pH and in 1 mM GSH at neutral pH (7.2). After 24 h, the dsDNA concentration was quantified using a fluorescent dye assay (dsDNABR; Qubit, Invitrogen), and monitored with a Qubit 3.0 Fluorometer (Invitrogen, Life Technologies, Carlsbad, CA, United States), yielding a dsDNA concentration in μg/mL.

### Effect of pH on H_2_O_2_ Production by GSH

Quantification of H_2_O_2_ production by 10 and 30 mM GSH at its intrinsic pH (5.4 and 3.3, respectively) and at pH 7 ± 0.2 (buffered using NaOH) in 1 × PBS was analyzed using a Hydrogen Peroxide Assay Kit – (Fluorometric-Near Infrared) (Abcam, Australia) complemented with a fluorescence plate reader (Tecan infinite M1000 pro). The protocol used to measure H_2_O_2_ in this study was as published by the manufacturer (Abcam – Hydrogen Peroxide Assay Kit).

### Effect of H_2_O_2_ on MRAB Biofilm Viability

Multi-drug resistant *A. baumannii* biofilms were grown for 48 h in 96-well plates as described above. After 48 h, biofilms were washed once with 1 × PBS followed by treatment with different concentrations of 0, 5, 10, 25, and 50 μM H_2_O_2_ (prepared by diluting an aliquot of 30% (v/v) H_2_O_2_ in 1 × PBS) for 24 h, at 37°C and shaking at 150 rpm. After 24 h, treated biofilm supernatant was washed once with 1 × PBS followed by addition of 200 μL of 1 × PBS plus 15 μL resazurin 0.05% w/v solution, and incubated for a further 24 h, at 37°C, 150 rpm. After 24 h, the fluorescent intensity of the biofilm was determined at Ex_544__nm_ and Em_590__nm_ (Tecan infinite M1000 pro). The control comprised a 1 × PBS treated biofilm showing 100% viability and percentage decrease in biofilm viability in the treated samples was calculated with respect to this control.

### Effect of GSH and GSSG on MRAB Biofilm Viability at Neutral pH

Multi-drug resistant *A. baumannii* biofilms were grown for 48 h in 96-well plates as described above. After 48 h, biofilms were washed once with 1 × PBS followed by treatment with 30 mM GSH (pH 7 ± 0.2) and 30 mM GSH (pH 7 ± 0.2) + 4 μg/mL amikacin and also with 30 mM GSSG and 30 mM GSSG + 4 μg/mL amikacin for 24 h, at 37°C and 150 rpm. After 24 h, treated biofilms were analyzed for viability using the resazurin assay as described above.

### Colony Forming Unit (CFU) Count of MRAB

Biofilms were treated with either GSH, antibiotics, or a combination of both, for 24 h as described above. Biofilms were then washed once with 1 × PBS and thoroughly homogenized by pipetting, with 200 μL of 1 × PBS. To establish a CFU count, 100 μl of homogenized suspension from each well was added to 900 μl of 1 × PBS (final volume 1 ml). The biofilm suspension was then serially diluted in 1 × PBS and 100 ml aliquots were spread on TSB plates and incubated for 24 h at 37°C. After 24 h the colonies on the plates were counted and numbers expressed as CFU/ml.

### Human Foreskin Fibroblast (HFF-1) Cell Culture

The HFF-1 cell line (ATCC-SCRC-1041) was cultured in DMEM, supplemented with 12% (v/v) FBS, 100 IU/ml penicillin and 100 μg/ml streptomycin. HFF-1 cells maintained in a T-25 cell culture flask (Corning, United States) at 37°C in a 5% (v/v) CO_2_ atmosphere and harvested at 90% confluence using 0.12% trypsin-EDTA. Cells were collected by first quenching Trypsin 1:1 (v/v) with supplemented media and transferred to 15 ml Falcon tubes, followed by centrifugation (5 min, 2000×*g*, 20°C). The supernatant was aspirated and the cell pellet was suspended in supplemented DMEM media for further experiments.

### MRAB-3 Growth on Pre-confluence HFF-1 Cells

To study the effect of MRAB-3 on pre-confluence of HFF-1 cells, HFF-1 cells were cultured and harvested as above. After harvesting, cells were plated to a density of 6 ± 0.5 × 10^5^ cells/mL in six-well plates (Corning) and allowed to incubate for 72 h at 37°C in a 5% (v/v) CO_2_ atmosphere to a confluence of 90%. 100 μL of MRAB -3 (OD_600__nm_ = 0.1 ± 0.02) suspended in 1 × PBS was then introduced into the confluent HFF-1-containing media and the plates allowed to incubate for a further 24 h. Where indicated, HFF-1-containing media were also incubated with a different treatment of either 10 or 30 mM GSH, or 4 μg/mL amikacin alone, or a combination of 10 mM GSH + 4 μg/mL amikacin, either in the presence or absence of bacteria. After 24 h, the HFF-1 cells in the well plates were imaged using phase contrast microscopy (Zeiss, Axio, Germany) for growth appearance, adherence and confluence.

### Effect of GSH on MRAB-3 Adhesion and Biofilm Formation

MRAB-3 was cultured and harvested as above and after harvesting at a bacterial density (OD_600__nm_ = 0.1 ± 0.02) was re-suspended in TSB and added to six-well plates (Corning Corp. United States) for incubation (24 h, at 37°C and 150 rpm) to initiate bacterial adhesion and biofilm growth. Where indicated, biofilm growth was also initiated in both presence and absence of 5, 10, and 30 mM GSH. After 24 h, the biofilm was washed once with 1 × PBS and imaged using phase contrast microscopy (Zeiss, Axio, Germany), for bacterial adhesion and colonization. Bacterial cells adherent to the surface of six-well plates were enumerated using ImageJ, where number of maxima present were quantified as explained on the ImageJ process menu, on its website^3^. The maxima were enumerated as this provided a more accurate presentation of individual cells clumped together, in comparison to simple thresholding.

### Statistical Method

All statistical analysis in the manuscript were done using Graphpad prism Unpaired *t-*test. The results are considered statistically significant if “*P* < 0.05”.

## Results

### GSH Changes the pH of TSB and 1 × PBS

Table 2 shows that addition of GSH materially decreased the pH of both TSB and 1 × PBS solution. The initial pH of both TSB and 1 × PBS was neutral (pH 7.18 and 7.41, respectively). After addition of 10, 20, or 30 mM GSH, the pH of TSB declined gradually to 6.45, 5.59, and 4.77, respectively. In the case of 1 × PBS, the pH dropped much further, to 5.51 at (10 mM GSH), 3.89 (20 mM GSH) and to pH 3.21 (30 mM GSH).

### Effect of GSH on Planktonic Growth of Clinical Bacterial Isolates

**FIGURE 1:**
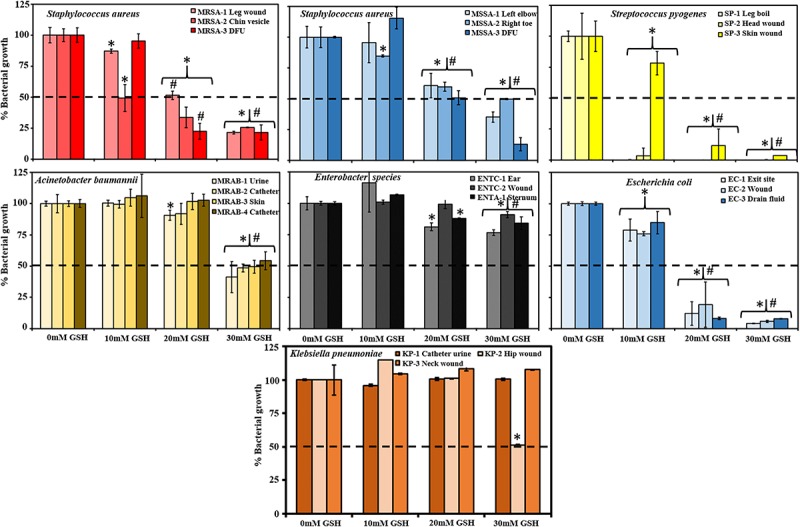
Effect of Glutathione (GSH) on growth of bacterial species isolated from Sydney hospitals. GSH showed concentration-dependent effects in inhibiting bacterial growth. 10 mM GSH did not affect bacterial growth for most of the isolates whereas; 20 mM GSH showed around 50% decrease in bacterial growth for all isolates of Methicillin-resistant *Staphylococcus aureus* (MRSA) and Methicillin-sensitive *S. aureus* (MSSA) and a less then 25% decrease for one *S. pyogenes* and *E. coli*. 30 mM GSH is very effective inhibiting greater than 50% growth with almost complete inhibition in case of *S. pyogenes* and *E. coli* and resistance in case of *Enterobacter* species and *K. pneumoniae*. Data represent mean ± SD; *n* = 4 experiments performed in biological replicates. Dotted line (- - -) in each graph indicates 50% bacterial growth. ^∗^*P* < 0.05 (statistically significant) when compared to control, #*P* < 0.05 (statistically significant) when compared to 10 mM GSH.

Figure 1 shows the effect of GSH (intrinsic pH) on growth of clinical isolates over 48 h. Bacterial growth in absence of GSH (control) is always considered as 100% growth and treatment growth levels are compared with respect to control. At 30 mM GSH, most bacterial species showed growth ≤50% after 48 h. Gram-positive species: MRSA and MSSA recorded growth of 22–27% and 13–50%, respectively, whereas *S. pyogenes* growth was almost completely inhibited (0–4%) at 30 mM GSH. Gram-negative species: For *A. baumannii* isolates, growth varied between 41 and 54%, Enterobacter sp. (77–91%), *E. coli* (4–8%) and *K. pneumoniae* (52–100%). At lower concentrations (10 and 20 mM GSH), only MRSA, *S. pyogenes* and *E. coli* showed growth ≤50%. These decreases in bacterial growth specifically at 30 mM GSH were statistically significant (*P* < 0.05) when compared to the control and 10 mM GSH-treated condition.

### MBC of *A. baumannii* Isolates in Presence of Amikacin

Table 3 and Supplementary Figure S2 show the MBC of the MRAB isolates. For MRAB-1 (Urine), MRAB-3 (skin) and MRAB-3 (catheter) the MBC was 16 μg/ml, whereas, for the MRAB-2 (catheter) isolate the MBC was 15 μg/ml (Table 3). In comparison to the control, MRAB isolates showed significant (*P* < 0.05) decreases in biofilm viability at all concentrations of amikacin, except for MRAB-4 (catheter isolate), which showed a significant decrease starting at 10 μg/ml amikacin (Supplementary Figure S2).

**TABLE 3:** Minimum biofilm inhibitory concentration (MBC) of multidrug resistant isolates of *Acinetobacter baumannii* (MRAB) in the presence of Amikacin.

***Acinetobacter baumannii***	**Amikacin (μg/ml)**
MRAB-1 Urine	16
MRAB-2 Catheter	15
MRAB-3 Skin	16
MRAB-4 Catheter	16

### Effect of GSH and Antibiotics on Bacterial Biofilm Viability

**FIGURE 2:**
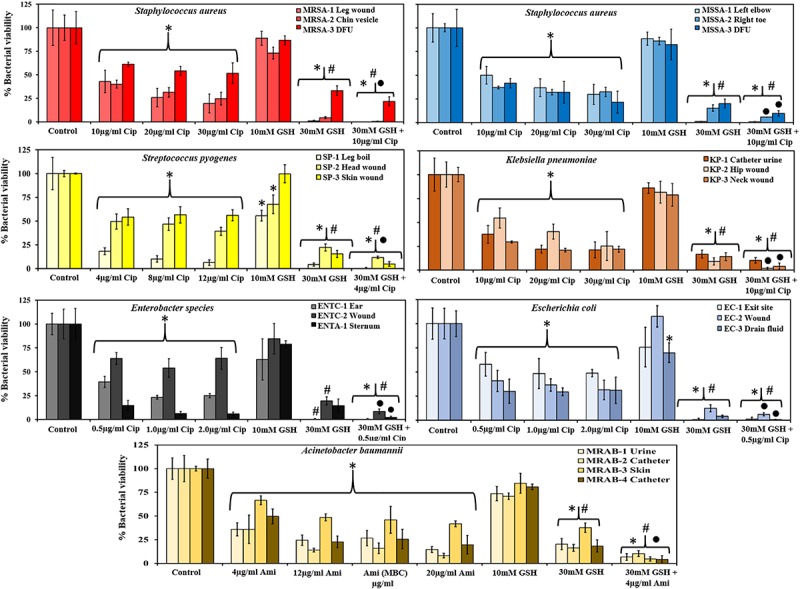
GSH reduces biofilm viability and enhances antibiotic efficiency. GSH showed a concentration-dependent effect in reducing biofilm viability. Ciprofloxacin (1–3 × MIC) significantly reduced biofilm viability of MRSA, MSSA, *S. pyogenes*, *Enterobacter* sp., *E. coli* and *K. pneumoniae.* For *A. baumannii* amikacin (1–5 × MIC) significantly reduced biofilm viability when compared to control. 10 mM GSH did not have any effect on biofilm viability, but 30 mM GSH significantly reduced biofilm viability of all bacterial species. A combination of 30 mM GSH and antibiotic of choice further reduced biofilm viability. ^∗^*P* < 0.05 when compared to control, #*P* < 0.05 when compared to antibiotic at 1 × MIC and ^∙^*P* < 0.05 when compared to 30 mM GSH. Data represent the mean ± SD of *n* = 4 experiments performed in biological replicate.

Figure 2 shows the effect of antibiotics and GSH on 48 h biofilms. Amongst Gram-positive bacteria, ciprofloxacin (MIC concentration 10 and 4 μg/ml for *S. aureus* MRSA and MSSA, and *S. pyogenes*, respectively) showed significant decreases in biofilm viability (∼40–61% and 37–50% for MRSA/MSSA and 18–54% for *S. pyogenes*, respectively) in comparison to untreated controls. An increase in ciprofloxacin concentration: to 30 μg/ml (*S. aureus*) and 12 μg/ml (*S. pyogenes*) showed further decreases in biofilm viability for few isolates: an average of 25% for strains MRSA-1 MRSA-2 and MSSA-3, and 6% for *S. pyogenes* (SP-1). Treatment with GSH at biological concentration (10 mM) did not show any significant decrease in biofilm viability in any isolates of MRSA and MSSA. However, *S. pyogenes* (SP-1) (56%) and SP-2 (68%) were more sensitive to GSH and showed a significant decrease in biofilm viability (*P* < 0.05). At a GSH, concentration of 30 mM an increase in biofilm disruption amongst all Gram-positive isolates was observed. For MRSA and MSSA, biofilm viability at 30 mM GSH ranged between 1–33% and 1–20%, respectively, and for *S. pyogenes* isolates, between 4 and 22%. Combination treatment comprising 30 mM GSH and 10 μg/ml ciprofloxacin showed further significant (*P* < 0.05) decreases in biofilm viability in all MRSA strains (0–22%). For MSSA (1–10%), there was a significant decrease only for strains MSSA-2 and MSSA-3, and significant decreases in for all *S. pyogenes* strains (0–12%).

Amongst the Gram-negative bacterial isolates, Enterobacter sp., and *E. coli* were found to have the lowest ciprofloxacin MIC (0.5 μg/ml) while for *K. pneumoniae*, the ciprofloxacin MIC was 10 μg/ml. At their respective MIC, biofilm viability of all isolates of Enterobacter sp. (15–64%), *E. coli* (30–58%), and *K. pneumoniae* (29–55%) showed significant decrease in viability compared to the control. However, further increases in ciprofloxacin concentration (to 2 μg/ml) did not result in significant decreases in viability for Enterobacter sp., and *E. coli*. On the other hand, all *K. pneumoniae* strains showed a large decrease in biofilm viability (21–25%) at 30 μg/ml ciprofloxacin, with statistical significance (*P* < 0.05) for *K. pneumoniae* (KP-3) neck wound isolate. In the case of amikacin sensitive *A. baumannii* isolates, treatment at the MIC (4 μg/ml) of amikacin showed a significant (*P* < 0.05) decrease in biofilm viability (36–67%). A further significant (*P* < 0.05) decrease in biofilm viability (8–42%) observed when amikacin concentration increased to 20 μg/ml.

A biological GSH concentration (10 mM) did not show any significant decrease in biofilm viability in most of the Gram-negative isolates. This is in contrast to use of the highest GSH concentration (30 mM), which showed a drastic (significant, *P* < 0.05) decrease in biofilm viability for all clinical isolates including *A. baumannii* (16–38%), Enterobacter sp. (0–20%), *E. coli* (1–12%) and *K. pneumoniae* (9–17%). Combined treatment with 30 mM GSH the + MIC concentration of antibiotics showed significant decreases (*P* < 0.05) in biofilm viability for most of the isolates: *A. baumannii* (4–10%), Enterobacter sp. (0–9%), *E. coli* (1–6%) and *K. pneumoniae* (2–10%) when compared to treatment with MIC concentration antibiotic or 30 mM GSH alone.

### Effect of GSH and Amikacin on MRAB Biofilm Biomass

**FIGURE 3:**
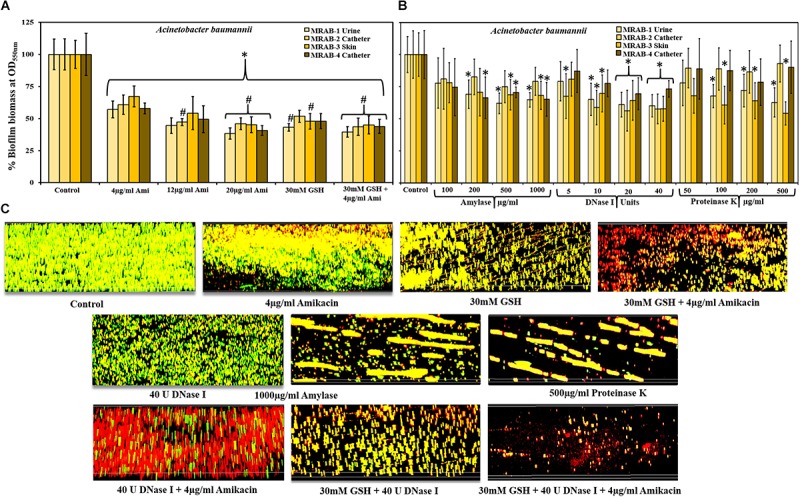
Effect of GSH, antibiotics and enzymes on MRAB biofilm biomass and biofilm architecture. **(A)** Biofilm biomass of MRAB isolates measured using the crystal violet assay showed statistically significant decreases in biomass when treated with amikacin, GSH alone or in combination, compared to untreated control. Amikacin at 1 × MIC reduced biomass to 58–67% whereas at 5 × MIC, biomass was reduced to between 38 and 46% for all isolates. 30 mM GSH decreased biomass to 43–52%, while a combination of GSH + amikacin decreased biomass by 40–45% for all isolates. **(B)** Both 20 and 40U DNase-I significantly reduced biofilm biomass (56–73% for all MRAB isolates). At higher concentrations of amylase (500 and 1000 μg/ml) and Proteinase K (200 and 500 μg/ml) treatment reduced biofilm biomass by 61–79% and 54–93%, respectively. **(C)** Biofilm architecture of MRAB-3 imaged using CLSM and complemented with Live/dead bacterial viability stain, showed a marked and distinct type of disruption in biofilm architecture when treated singly with amikacin, GSH, or enzyme, or combinations thereof. In panels **(A,B)** ^∗^*P* < 0.05 when compared to control and #*P* < 0.05 when compared to 4 μg/ml amikacin. Data represent the mean ± SD of *n* = 4 experiments performed in biological replicate.

Crystal violet assays measured biofilm biomass of MRAB isolates and showed a statistically significant decrease in biomass when treated with 4–20 μg/ml amikacin or 30 mM GSH alone or in combination, compared to untreated biofilm (Figure 3A). With MIC concentration (4 μg/ml) amikacin recorded 57–67% biofilm biomass whereas, at the highest concentration of amikacin used in this study (20 μg/ml) recorded only 38–46% biomass. When treated with 30 mM GSH, the biomass percentage ranged between 43 and 52%, however, when compared to 4 μg/ml amikacin, only MRAB-1 and MRAB-2 showed a significant decrease (*P* < 0.05). Interestingly, a combination of 30 mM GSH + 4 μg/ml amikacin showed statistically significant decreases in biomass (40–45%) for all isolates in comparison to 4 μg/ml amikacin alone.

### Enzymatic Treatment on MRAB Biofilm

We also investigated the effect of DNase-I, amylase and Proteinase K on all MRAB biofilm biomass using CV assays (Figure 3B). In comparison to the control, DNase-I-treated MRAB biofilms for all strains at 20 and 40U showed a statistically significant reduction in biofilm biomass (biomass recorded as 56–73%) whereas, at low concentration (5U) only MRAB-2 and at 10U DNase I only MRAB-1, 2, and 3 showed a significant reduction in biomass. When treated with 500 and 1000 μg/ml amylase, the biomass ranged between 61 and 79%, with significant reductions for MRAB-1, MRAB-2, and MRAB-3. With 200 μg/ml amylase, only MRAB-1 and MRAB-4 showed a significant reduction whereas, 100 μg/ml amylase did not result in biomass reduction. Treatment with 100, 200, and 500 μg/ml Proteinase K resulted in a biomass between 54 and 93% of control, and a significant difference was observed only for the urine and skin isolates MRAB-1 and -3.

### Amikacin, GSH and Enzymes Modulate MRAB-3 Biofilm Architecture

The biofilm architecture of MRAB-3 changed significantly when subjected to different treatments Figure 3C. The effect of 4 μg/ml amikacin, 30 mM GSH, 40U DNase-I, 1000 μg/ml amylase and 500 μg/mL Proteinase K, individually or in combination (two or three components) on established biofilms of MRAB-3 is shown in Figure 3C. CLSM complemented Live/dead biofilm imaging and showed marked disruption in the biofilm architecture of MRAB when treated for 24 h with GSH or enzymes. The DNase-I treated biofilm showed considerably different biofilm architecture when exposed to other enzymes (amylase and Proteinase K). However, treatment with amikacin alone did not disrupt the biofilm, but enhanced more dead (red) biofilm cells than in the corresponding control. Conversely, for combinations of GSH + amikacin, DNase-I + amikacin, GSH + DNase-I and GSH + DNase-I + amikacin, a larger increase in biofilm disruption and more modulated changes in biofilm architecture were visible than in the corresponding individual treatment regimens and control. A comparison of the percentage of live/dead cells in biofilm showed an increase in live percentage for the control (∼85%), whereas, treated biofilms showed a decrease in live biofilm of between 39 and 73%. A statistically significant difference was observed for amikacin, GSH, the combination of GSH + amikacin, Proteinase K, DNase I + amikacin, GSH + DNase I and GSH + DNase I + amikacin (Supplementary Figure S3).

### Modulation and Cleavage of dsDNA Due to GSH Acidity

**FIGURE 4:**
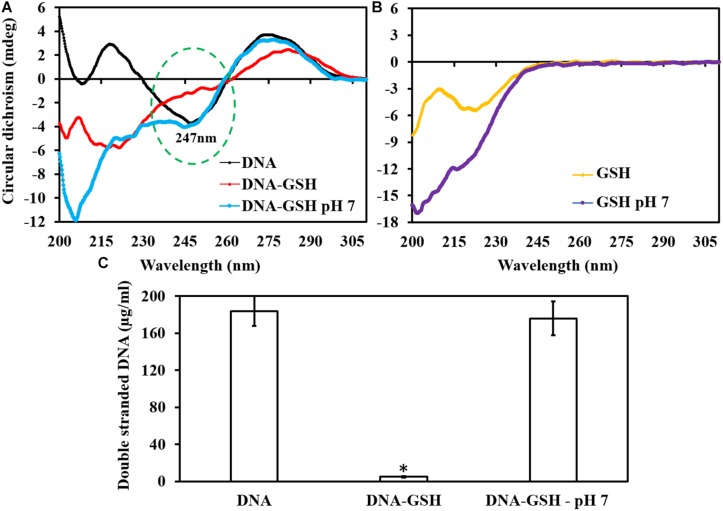
Effect of GSH on dsDNA. The circular dichroism curve showed dsDNA modulation at its sugar phosphate backbone. **(A)** dsDNA has a peak at 247 nm at intrinsic pH, and no change occurs when it is exposed to GSH at pH 7.2. **(B)** Intrinsic and buffered GSH curve showed a peak at around 220 nm. **(C)** Qubit fluorometer quantification of dsDNA showed significant reduction in dsDNA concentration when exposed to GSH at intrinsic pH. ^∗^*P* < 0.05 when compared to control and GSH at neutral pH.

Circular Dichroism peaks clearly indicated that GSH at its intrinsic pH drastically modulates the DNA sugar phosphate backbone (peak 247 nm) whereas GSH at neutral pH did not alter this peak (Figure 4A). GSH by itself does not have a peak at or near 247 nm, but rather peaks at around 220 nm (Figure 4B). Qubit fluorometer quantification of dsDNA showed that GSH at intrinsic pH reduced dsDNA concentration to 5 μg/ml in comparison to 176 μg/ml (GSH at neutral pH) and 184 μg/ml for control/untreated (Figure 4C).

### Effect of GSH at Neutral pH on MRAB Biofilm Viability

**FIGURE 5:**
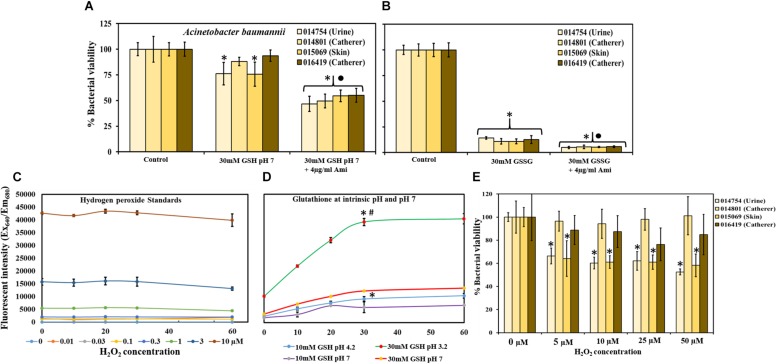
Effect of GSH, GSSG and H_2_O_2_ at neutral pH on MRAB biofilm viability. **(A)** 30 mM GSH (buffered to pH 7) resulted in a small biofilm viability decrease (76–94% viable), while combination with amikacin enhanced the decrease significantly (47–55% viable) when compared to both control and 30 mM GSH (pH 7) alone. **(B)** 30 mM GSSG alone and in combination with 4 μg/ml amikacin significantly decreased MRAB biofilm viability to 11–14% and 5–6%, respectively, when compared to control. **(C,D)** Standard H_2_O_2_ data used to determine H_2_O_2_ production by 10 and 30 mM GSH in 1 × PBS at intrinsic pH, and at buffered neutral pH 7. At intrinsic pH, GSH produced significantly more H_2_O_2_ (higher fluorescent intensity) than at its corresponding neutral pH concentration. **(E)** H_2_O_2_ treatment showed variation in MRAB biofilm viability among isolates. When compared to control only, MRAB-1 and MRAB-3 isolates showed statistically significant decreases in biofilm viability at all H_2_O_2_ concentrations. However, for MRAB-2 and MRAB-4, biofilm viability was similar to control at all H_2_O_2_ concentrations. ^∗^*P* < 0.05 compared to control ^∙^*P* < 0.05 when compared to 30 mM GSH **(A)** and 30 mM GSSG **(B)** and #*P* < 0.05 when compared to all other conditions **(D)**. Data represent the mean ± SD of *n* = 4 experiments performed in biological replicate.

Figure 5A shows the effect of 30 mM GSH (buffered to pH 7), alone and in combination with 4 μg/ml amikacin on MRAB biofilm viability. At neutral pH, GSH showed only small decreases in MRAB biofilm viability (76–94%) with statistically significant decreases only for MRAB-1 and MRAB-3 in comparison to control. When combined with amikacin, biofilm viability decreased (47–55%) significantly (*P* < 0.05) when compare to both control and 30 mM GSH (pH 7) alone treatment.

### Effect of GSSG on MRAB Biofilm Viability

MRAB biofilms subjected to treatment with 30 mM GSSG at intrinsic pH showed statistically significant decreases in biofilm viability (11–14%) when compared to control biofilms. Further significant decreases of 5–6% in MRAB biofilm viability compared to GSSG alone treatment were observed when biofilms were exposed to 30 mM GSSG + 4 μg/ml amikacin (Figure 5B).

### The Influence of pH on H_2_O_2_ Production by GSH

Figure 5C showed that fluorescent intensity corresponds to standard H_2_O_2_ concentration, as measured using a H_2_O_2_ assay kit. Standard H_2_O_2_ data were used to determine H_2_O_2_ production by GSH at intrinsic pH i.e., 10 mM = pH 5.51 and 30 mM = pH 3.2, and at buffered neutral pH 7. In general, at intrinsic pH GSH produced a significantly higher concentration H_2_O_2_ (higher fluorescent intensity) than at its corresponding neutral pH (*P* < 0.05). 30 mM GSH (intrinsic pH) showed 3 and 4 fold increases in H_2_O_2_ production in comparison to 30 mM GSH (pH 7) and 10 mM GSH (at intrinsic pH), respectively, (Figure 5D).

### H_2_O_2_ Impact on MRAB Biofilm Viability

Multi-drug resistant *A. baumannii* biofilms, when subjected to H_2_O_2_ treatment, showed variations in biofilm viability among isolates. MRAB-1 and MRAB-3 (urine and skin isolates) showed a statistically significant decrease in biofilm viability, to 53 and 58%, respectively, at 50 μM H_2_O_2_ (*P* < 0.05). However, for the catheter isolates (MRAB-2 and MRAB-4), biofilm viability was similar to control at all H_2_O_2_ concentrations (Figure 5E).

### Post-treatment CFU Counts of MRAB Biofilms

**FIGURE 6:**
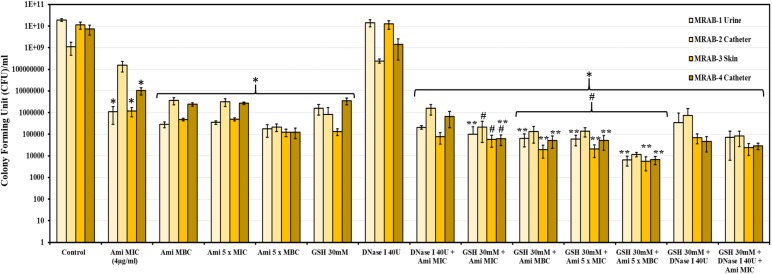
CFU count of MRAB biofilms after GSH, amikacin and DNase-I treatment: CFU/ml of control, amikacin, DNase-I and GSH of MRAB isolates was log_10_ 9–10.2, log_10_ 5.1–8.2, log_10_ 8.3–10.2 and 5.1–6.5, respectively. Combination treatments comprising: DNase-I + amikacin, GSH + amikacin and GSH + DNase-I on biofilms resulted in a CFU/ml of log_10_ 4.9–6.2, 3.7–5.3, and 4.6–5.9, respectively. The triple combination of GSH + DNase-I + amikacin decreased the CFU/ml to log_10_ 4.3–4.9. Data represent the mean ± SD of *n* = 3 experiments performed in biological replicate. ^∗^*P* < 0.05 compared to control, #*P* < 0.05 when compared to amikacin vs. GSH + amikacin and ^∗∗^*P* < 0.05 when compared to GSH vs. GSH + amikacin.

Control/untreated biofilms and DNase-I-treated biofilms had a CFU/ml at log_10_ of 9-10.2. The CFU/ml of amikacin (4–80 μg/ml) and GSH (30 mM)-treated biofilms were log_10_ 5.1-8.2 and 5.1-6.5, respectively. Double combination treatments comprising: DNase-I (40U) + amikacin (4 μg/ml), GSH (30 mM) + amikacin (4–80 μg/ml) and GSH (30 mM) + DNase-I (40U) resulted in decreased CFUs/ml of log_10_ 4.9–6.2, 3.7–5.3 and 4.6–5.9, respectively. GSH (30 mM) + amikacin (MBC) showed the most effective decrease in CFU/ml (1og_10_ 3.7-4.1). Whereas, triple combination treatment (30 mM GSH + 40U DNase-I + 4 μg/ml amikacin) resulted in a CFU/ml between log_10_ 4.3–4.9. For all treatment conditions (except for DNase I alone treatment) the decreases in CFU/ml were statistically significant when compared to 1 × PBS treated control (*P* < 0.05). In addition, when treatment with amikacin alone was compared with GSH + amikacin, the reduction in CFU/ml in combination treatment was significant, (*P* < 0.05) especially at higher amikacin concentrations (Figure 6).

### GSH Fosters HFF-1 Confluence and Inhibits MRAB Colonization

**FIGURE 7:**
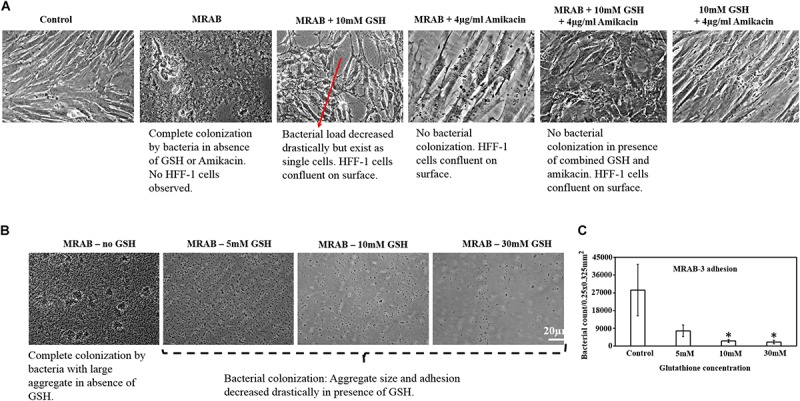
Effect of GSH on MRAB colonization and HFF-1 confluence. **(A)** Pre-confluent HFF-1 cells were completely disrupted when exposed to MRAB-3, whereas GSH, amikacin alone or in combination, inhibited MRAB colonization and retained HFF-1 cell confluence. **(B,C)** GSH alone was shown to significantly decrease MRAB-3 adhesion and colonization. ^∗^*P* < 0.05 compared to control and data represent the mean ± SD of *n* = 3 experiments performed in biological replicate.

Figure 7A shows that MRAB infected and completely removed pre-confluence HFF-1 cells. Treatment with GSH or amikacin alone drastically reduced MRAB adhesion and colonization and concurrently induced a greater than 50% HFF-1 increase in adhered cells compared to MRAB-alone infected HFF-1. The combination of GSH + amikacin showed complete inhibition of MRAB colonization and maintenance of HFF-1 cell confluence similar to the control. GSH alone in a concentration dependent manner hindered MRAB-3 adhesion and colonization on surfaces, with 30 mM GSH showing the greatest reduction in bacterial adhesion (Figure 7B). Quantification of MRAB-3 adhesion on surfaces showed approximately 2.7 × 10^4^ bacteria per 0.25 × 0.325 mm^2^ (area) for the 1 × PBS treated control whereas, in the presence of GSH bacterial adhesion decreased significantly (>5 × 10^3^ bacteria) especially at 10 and 30 mM GSH (Figure 7C).

## Discussion

Glutathione in naturally present in mammalian cells. However, its biological concentration varies considerably. Intracellular fluid (cytosol) has up to 10 mM of GSH whereas the plasma GSH concentration is very low (micromolar range) (Forman et al., 2009). In cystic fibrosis, the efficiency of intrinsic GSH synthesis is very low compared to healthy populations and studies have demonstrated that GSH deficiency leads to recurrent bacterial infections (Roum et al., 1993; Ristoff et al., 2001; Ghezzi, 2011). An interesting feature of GSH revealed in this study is the intrinsic acidity of GSH, and how this inhibits bacterial growth and disruption of biofilms (Figures 1, 2). At biological levels (10 mM), GSH had no disruptive impact on the bacterial species tested with the exception of for *S. pyogenes*, whereas at 30 mM GSH, a greater than 50% decrease in bacterial growth for most bacterial species was observed, with *S. pyogenes* growth nearly completely inhibited. Our findings were corroborated by a previous study (Schairer et al., 2013), which also reported that concentration-dependent GSH mediated acidity (pH < 4) inhibits bacterial growth. Interestingly, the Gram-negative species *K. pneumoniae* and *Enterobacter* sp. used in this study were highly resistant to 30 mM GSH (Figure 1). This could potentially be due to bacterial ability to confront low pH/acid via mechanisms such as removal of H^+^ via a proton pump, production of enzymes and proteins to repair degraded proteins and DNA, modulation in cell envelope, and changes in cell density (Cotter and Hill, 2003).

30 mM GSH showed excellent levels of disruption and killing, with a greater than 50% reduction in biofilm viability for all bacterial species used in this study (*P* > 0.05, for all isolates). Reduction in biofilm viability achieved by treatment with 30 mM GSH alone is either analogous to or improved in comparison to maximum antibiotic concentrations (3, 4, and 5 × MIC) used in this study. Most remarkably, for almost all bacterial isolates, 30 mM GSH was shown to enhance antibiotic efficiency, indicating the practicality of GSH in treatment of biofilm-associated infections (Figure 2). Our study showed that the MBC of amikacin for the MRAB isolates tested was about four fold higher compared to its MIC (4 μg/ml) (Table 3 and Supplementary Figure S2). MRAB biofilm treated with 30 mM GSH alone and GSH + amikacin (1 × MIC) demonstrated enhanced reduction in biomass compared to 1 × MIC amikacin alone (Figure 3A). In general, in all treated conditions a drastic modulation of the MRAB biofilm architecture was evident, with enhanced disruption and an increase in dead (red) biofilm cells (Figure 3C and Supplementary Figure S3).

Biofilm disruption by GSH acidity is triggered through cleaving and destabilization of biopolymers such as extracellular DNA (eDNA), polysaccharides and proteins. By using CD and Qubit fluorometry, we confirmed that acidic GSH cleaves dsDNA and destabilizes its double helix structure (Figure 4). A significant change in pH affects the structure of all macromolecules, for instance, presence of a higher proportion of H^+^ or low pH solution cleaves DNA double helix strands by protonating the DNA phosphodiester groups and consequently destabilizing the hydrogen bonds linking DNA nitrogenous bases guanine and cytosine (G-C) (Sorokin et al., 1986). Similarly, low pH triggers acid-induced protein unfolding and degradation via hydrolysis and aggregation (Estey et al., 2006). Bacterial cells exposed to acidic pH undergo substantial structural damage to their cell wall, and intracellular macromolecules such as DNA and proteins (Cotter and Hill, 2003). Rao et al. (1984) showed that at low pH (4–6), bacteria undergo significant modulation in their cytological characteristics, physiological activity and decline in population. In contrast, Dwyera et al. (2014) claim that pre-treatment of bacteria (*E. coli*) with antioxidants (GSH and Ascorbic acid), reduces antibiotic-mediated reactive oxygen species production and consequently limits antibiotic lethality.

The role of biopolymers in MRAB biofilm integration was also revealed by the enzymatic part of the treatments, which resulted in a significant reduction in biofilm biomass and distinctive disruption in biofilm architecture. DNase-I and amylase treatment confirmed, through total loss of structure, that eDNA and polysaccharides are the common biopolymers responsible for MRAB biofilm matrix integration. Interestingly, MRAB urine and skin isolates must contain significant levels of protein integrated within their biofilms, as evidenced by Proteinase K treatment (Figure 3B). This assumption was confirmed through CLSM images of MRAB-3 (skin isolate) and provided further evidence of the distinct modulation of its biofilm architecture after being subjected to different enzyme solutions. DNase-I treatment showed more single colonies uniformly distributed on the surface, whereas CLSM images revealed amylase and Proteinase treatment showed a mixture of small and big clusters in biofilm architecture (Figure 3C). The combination of DNase-I + amikacin resulted in a strong increase in dead biofilm compared to DNase I treatment alone, and the triple therapy combination of GSH + DNase-I + amikacin showed the highest level of biofilm disruption (Figure 3C). Results from this study confirm that MRAB biofilm matrix integration is significantly dependent on the biopolymers present, and this finding aligns with previous studies, which reported that eDNA, polysaccharides and proteins play an essential role in *A. baumannii* biofilm formation and stability (Choi et al., 2009; Kari et al., 2011; Sahu et al., 2012; Gawande et al., 2014). By allowing the biopolymers to directly bind to antibiotics and inhibiting antibiotic penetration into the biofilm, the matrix acts as a shield for the bacteria concealed inside it (Lewis, 2001; Wilton et al., 2016; Clayton and Thien-Fah, 2017).

The efficacy of GSH is enhanced by its intrinsic acidity (pH 3.21 in 1 × PBS and 4.77 in TSB) at 30 mM GSH. Buffering of 30 mM GSH (in TSB and 1 × PBS), with pH to neutral (7) had no impact on bacterial growth (data not shown) and very minimal impact on biofilm viability (Figure 5A). On the other hand, addition of 30 mM GSSG, an oxidized form of GSH which has no antioxidant properties but is intrinsically acidic, resulted in a reduction in biofilm viability comparable to or greater than 30 mM GSH at intrinsic pH levels (Figure 5B). This result indicates that GSH concentration is likely dependent on acidity and that its acidity plays a significant role in its biofilm disruption activity. Previous studies also reported that butyric acid (pH > 5) has a significantly inhibits bacterial growth and this could be possibly due to acidification of the bacterial cytoplasm and protonation-(H^+^)-triggered modulation of bacterial cell ATP synthesis (Sun et al., 1998).

The thiol group of GSH may be a significant factor in the biofilm disruption process. Studies of another thiol-containing antioxidant *N*-acetylcysteine (NAC), at its intrinsic pH, showed that it destabilizes the biofilm matrix by chelating divalent cations (e.g., Ca^2+^ and Mg^2+^), inhibition of bacterial extracellular polysaccharide production, modulation of bacterial cell physiology and the impact on its metabolism (Olofsson et al., 2003; Zhao and Liu, 2010; Blasi et al., 2016; Choid et al., 2018). Whether the thiol group plays a greater role in biofilm disruption than the acidity of GSH has yet to be determined. Nevertheless, results from our study clearly suggest that GSH-mediated biofilm disruption is negligible at neutral pH, and thus the acidity of GSH is a critical factor in the effectiveness of its biofilm disruption and reduction of bacterial viability.

Our investigation of H_2_O_2_ production by GSH during disruption and killing of MRAB biofilms produced interesting results (Figures 5C–E). Previous studies had reported that GSH undergoes auto-oxidation to produce H_2_O_2_ (Albro et al., 1986). In this study, we showed that H_2_O_2_ production is higher in acidic conditions, mainly due to the presence of more H^+^ ions (Figure 5D). GSH-mediated H_2_O_2_ production is likely to have had an impact on the biofilms from two of the four MRAB isolates. MRAB urine and skin isolates showed significant biofilm viability reductions of up to 40%, whereas catheter isolates did not display significant viability reductions (Figure 5E). MRAB are catalase positive and so it is not surprising to see a minimal effect of H_2_O_2_ on viability of their biofilms. Another interesting outcome was that GSH treatment did not change the efficiency of MRAB resistance to the antibiotics (Augmentin, ciprofloxacin, gentamicin and tobramycin) tested in this study (Supplementary Figures S1A–D), indicating that GSH does not influence MRAB’s gene expression profile, however, further research is needed to ascertain the validity of these findings. In parallel with the above observations, the use of GSH in a combination treatment of MRAB especially with an antibiotic (at highest concentration, 5 × MBC) resulted in the lowest recoverable CFU count (i.e., the lowest level of viability) (Figure 6). An additional advantage of GSH is that it helps in maintaining and fostering HFF-1 cell confluence while significantly inhibiting MRAB adhesion, colonization and HFF-1 infection (Figure 7). However, it should be noted that GSH at high concentrations (e.g., 30 mM) induces morphological changes in HFF-1 cells (Supplementary Figure S1E).

The thiol-based antioxidant NAC has undergone clinical trials for use in respiratory related infections (Schito et al., 2007; Macchi et al., 2012). However, in some cases GSH has an added advantage in comparison to NAC alone, because in groups such as the immunocompromised, those with cystic fibrosis and liver dysfunction, as well as the elderly, the capacity to synthesis GSH has already significantly diminished. This could potentially lead to an inability to convert cysteine or NAC into GSH (Schmitt et al., 2015). A study also showed that a novel sublingual form of GSH (Sublinthions by Laboratoires Le Stum, Larmor-Plage, France) enhances the bioavailability of GSH in plasma when compared to oral NAC supplementation (Schmitt et al., 2015).

In this study, we demonstrated that GSH acidity plays a key role in biofilm disruption of many MDR bacterial isolates and enhances antibiotic efficacy. With MRAB, biofilm integration is primarily dependent on eDNA and polysaccharides and the cleaving of these biopolymers enhances the antibiotic’s efficiency in killing the bacteria.

## Data Availability

The datasets generated for this study are available on request to the corresponding author.

## Author Contributions

TD designed and conducted the experiments and wrote the manuscript. DP designed and conducted the experiments. AM and JF conducted the experiments. GW, FK, TG, and JM designed the experiments and wrote the manuscript.

## Conflict of Interest Statement

GW, FK, and TG are employed by Whiteley Corporation. The remaining authors declare that the research was conducted in the absence of any commercial or financial relationships that could be construed as a potential conflict of interest.

## Footnotes

1 https://www.who.int/news-room/detail/27-02-2017-who-publishes-list-of-bacteria-for-which-new-antibiotics-are-urgently-needed

2 https://www.legislation.nsw.gov.au/inforce/dae79cae-8043-47df-9c37-fe8d1d7e38b3/1983-164.pdf

3 https://imagej.net/docs/guide/146-29.html#Flo:Maxima-outputs and https:// imagej.net/docs/menus/process.html#find-maxima

## Supplementary Material

FIGURE S1 Effect of different antibiotics on MRAB biofilm viability. MRAB biofilms subjected to Augmentin **(A)**, ciprofloxacin **(B)**, gentamicin **(C)** and Tobramycin **(D)** showed decreases in biofilm viability of 56–94%, 58–72%, 60–100% and 70–90%, respectively. **(A–D)** When antibiotics were combined with 30 mM GSH, MRAB biofilm viability decreased significantly to 14–27%. **(E)** Modulation in HFF-1 cell morphology observed when exposed to GSH 30 mM. For Panels **(A–D)**, ^∗^*P* < 0.05 compared to control, #*P* < 0.05 compared to the respective antibiotic concentrations. Data represent the mean ± SD of *n* = 4 experiments performed in biological replicate.

FIGURE S2 The MBC of amikacin on MRAB isolates. All MRAB isolates except MRAB-4 showed a significant decrease in their biofilm viability at the lowest amikacin concentration (4 μg/ml). MRAB-4 showed a significant reduction from 10 μg/ml amikacin **(A)**. In comparison to the control, growth of MRAB isolates in presence of amikacin were significantly reduced from 6 μg/ml amikacin for MRAB-1 and -2, and from 8 and 10 μg/ml amikacin for MRAB-3 and -4, respectively **(B)**. The MBC of all MRAB isolates are similar 15–16 μg/ml. ^∗^*P* < 0.05 compared to control, #*P* < 0.05 compared to 4 μg/ml amikacin and data represent the mean ± SD of *n* = 4 **(A)** and *n* = 3 **(B)** experiments performed in biological replicate.

FIGURE S3 Quantification of live/dead biofilm biomass of MRAB-3. The control showed the highest percentage of live biofilm (∼85%) in comparison to their treatment conditions. Treatment with amikacin and GSH alone showed approximately 54 and 56% live biofilm, while enzymatic treatment resulted in between 54 and 73% live biofilm. Triple combination treatment resulted in the least percentage of live biofilm (∼39%). ^∗^*P* < 0.05 compared to control. Data represent the mean ± SD of *n* = 3 experiments performed in biological replicate.
