# Supplementary figures and images for: Conditions Under Which Glutathione Disrupts the Biofilms and Improves Antibiotic Efficacy of Both ESKAPE and Non-ESKAPE Species
Source: Front Microbiol. 2019 Aug 30;10:2000. doi: 10.3389/fmicb.2019.02000 (PMC6730566; doi:10.3389/fmicb.2019.02000)

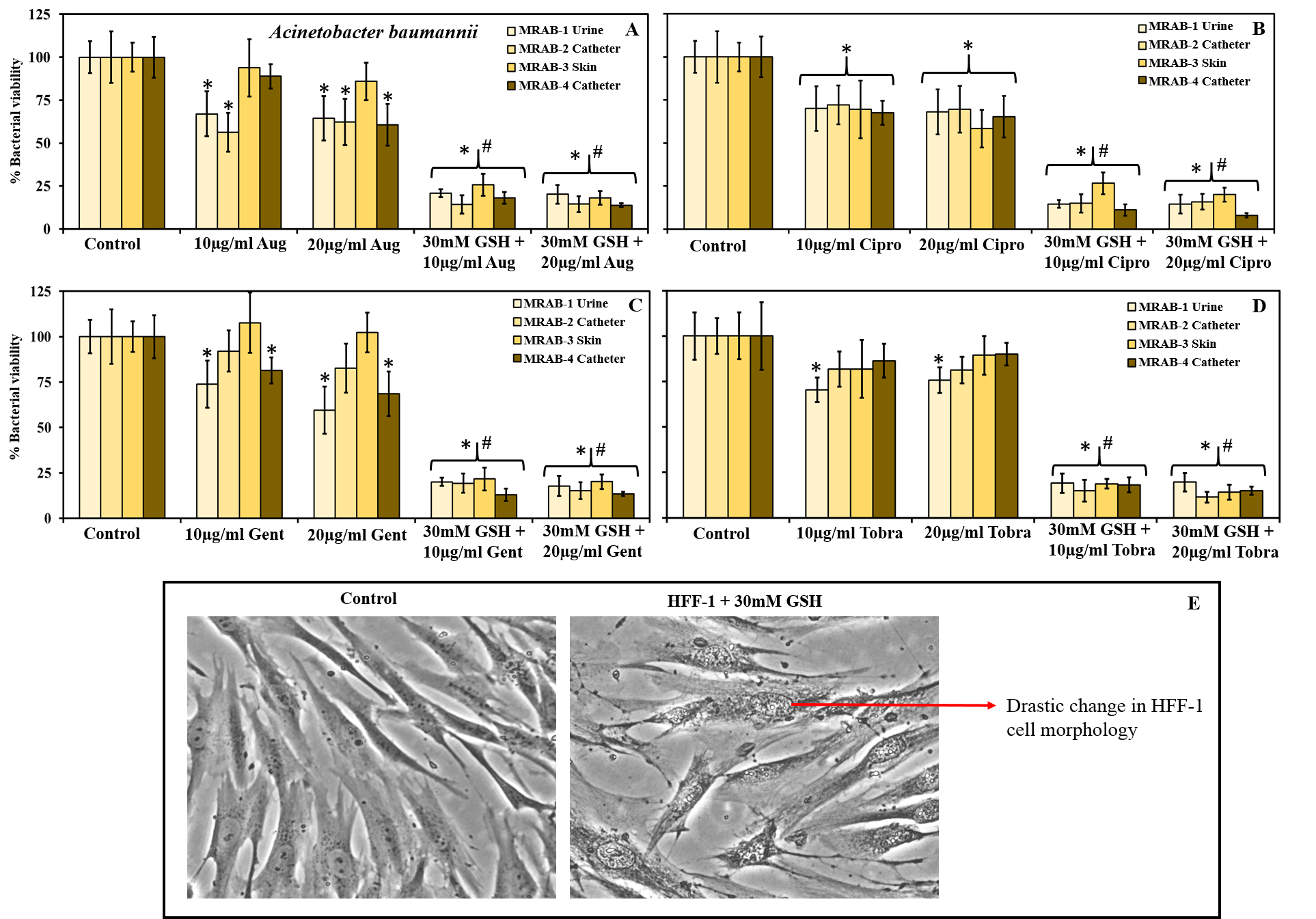

Supplement: FIGURE S1 — Effect of different antibiotics on MRAB biofilm viability. MRAB biofilms subjected to Augmentin (A), ciprofloxacin (B), gentamicin (C) and Tobramycin (D) showed decreases in biofilm viability of 56–94%, 58–72%, 60–100% and 70–90%, respectively. (A–D) When antibiotics were combined with 30 mM GSH, MRAB biofilm viability decreased significantly to 14–27%. (E) Modulation in HFF-1 cell morphology observed when exposed to GSH 30 mM. For Panels (A–D), ∗P < 0.05 compared to control, #P < 0.05 compared to the respective antibiotic concentrations. Data represent the mean ± SD of n = 4 experiments performed in biological replicate. [file Image_1.tif]

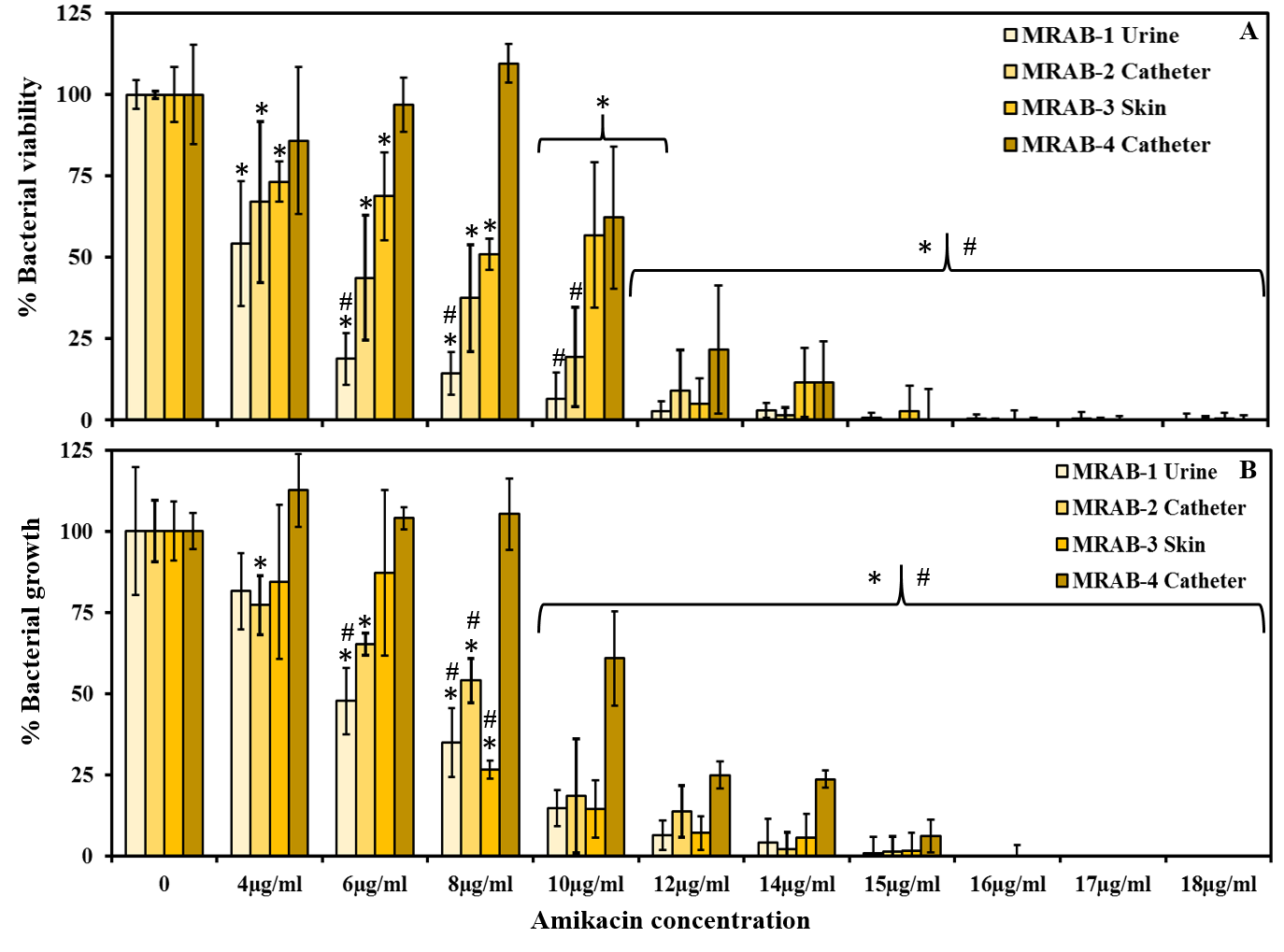

Supplement: FIGURE S2 — The MBC of amikacin on MRAB isolates. All MRAB isolates except MRAB-4 showed a significant decrease in their biofilm viability at the lowest amikacin concentration (4 μg/ml). MRAB-4 showed a significant reduction from 10 μg/ml amikacin (A). In comparison to the control, growth of MRAB isolates in presence of amikacin were significantly reduced from 6 μg/ml amikacin for MRAB-1 and -2, and from 8 and 10 μg/ml amikacin for MRAB-3 and -4, respectively (B). The MBC of all MRAB isolates are similar 15–16 μg/ml. ∗P < 0.05 compared to control, #P < 0.05 compared to 4 μg/ml amikacin and data represent the mean ± SD of n = 4 (A) and n = 3 (B) experiments performed in biological replicate. [file Image_2.tif]

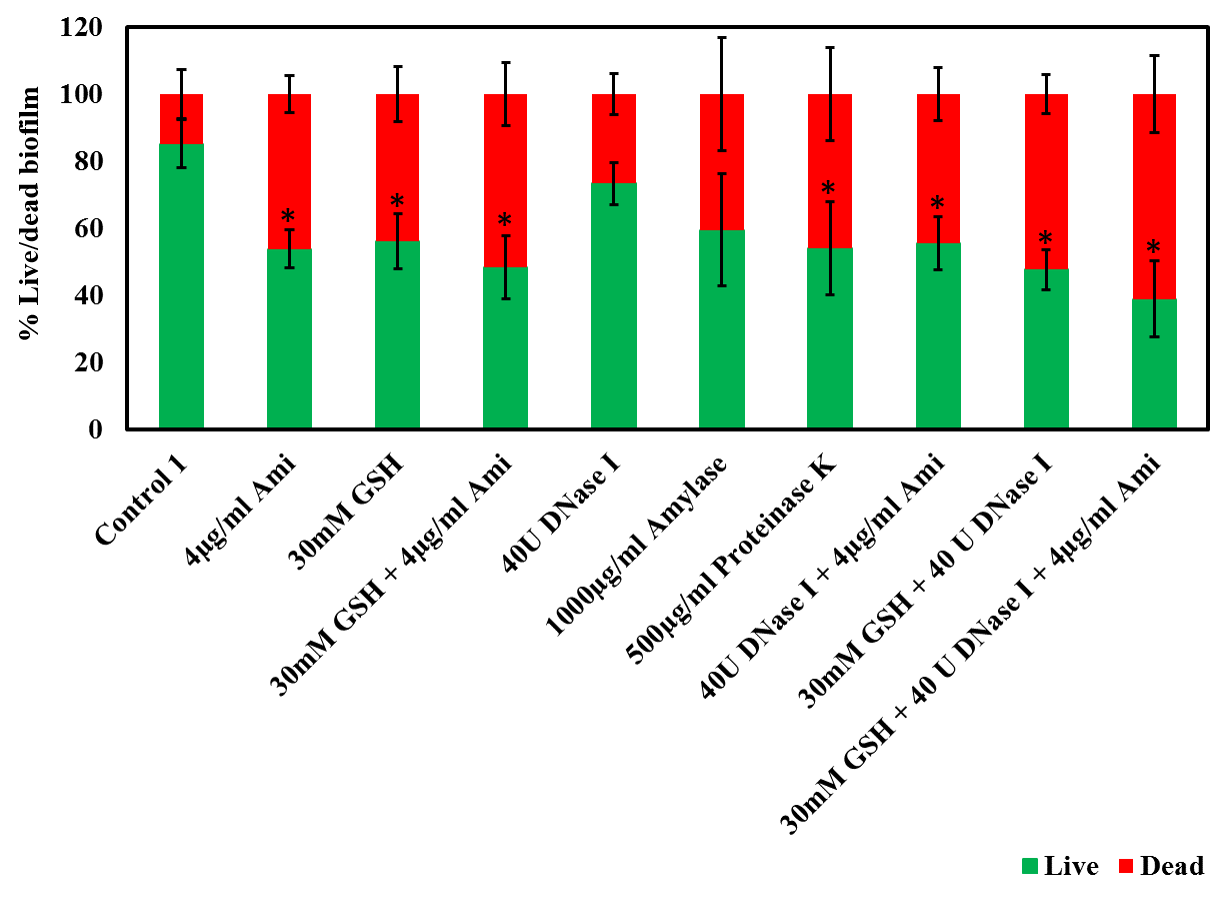

Supplement: FIGURE S3 — Quantification of live/dead biofilm biomass of MRAB-3. The control showed the highest percentage of live biofilm (∼85%) in comparison to their treatment conditions. Treatment with amikacin and GSH alone showed approximately 54 and 56% live biofilm, while enzymatic treatment resulted in between 54 and 73% live biofilm. Triple combination treatment resulted in the least percentage of live biofilm (∼39%). ∗P < 0.05 compared to control. Data represent the mean ± SD of n = 3 experiments performed in biological replicate. [file Image_3.tif]
